# Modeling toolchain for realistic simulation of photoacoustic data acquisition

**DOI:** 10.1117/1.JBO.27.9.096005

**Published:** 2022-09-14

**Authors:** Jan-Willem Muller, Mustafa Ü. Arabul, Hans-Martin Schwab, Marcel C. M. Rutten, Marc R. H. M. van Sambeek, Min Wu, Richard G. P. Lopata

**Affiliations:** a Eindhoven University of Technology, Photoacoustics and Ultrasound Laboratory Eindhoven, Department of Biomedical Engineering, Eindhoven, The Netherlands; b Catharina Hospital, Department of Vascular Surgery, Eindhoven, The Netherlands; c Cardiovascular Biomechanics Group, Department of Biomedical Engineering, Eindhoven, The Netherlands

**Keywords:** photoacoustic imaging, modeling, k-wave, Monte–Carlo, toolchain

## Abstract

**Significance:**

Physics-based simulations of photoacoustic (PA) signals are used to validate new methods, to characterize PA setups and to generate training datasets for machine learning. However, a thoroughly validated PA simulation toolchain that can simulate realistic images is still lacking.

**Aim:**

A quantitative toolchain was developed to model PA image acquisition in complex tissues, by simulating both the optical fluence and the acoustic wave propagation.

**Approach:**

Sampling techniques were developed to decrease artifacts in acoustic simulations. The performance of the simulations was analyzed by measuring the point spread function (PSF) and using a rotatable three-channel phantom, filled with cholesterol, a human carotid plaque sample, and porcine blood. *Ex vivo* human plaque samples were simulated to validate the methods in more complex tissues.

**Results:**

The sampling techniques could enhance the quality of the simulated PA images effectively. The resolution and intensity of the PSF in the turbid medium matched the experimental data well. Overall, the appearance, signal-to-noise ratio and speckle of the images could be simulated accurately.

**Conclusions:**

A PA toolchain was developed and validated, and the results indicate a great potential of PA simulations in more complex and heterogeneous media.

## 1 Introduction

Photoacoustic (PA) imaging is a relatively new biomedical imaging modality that employs the wide variety of optical properties found in tissue to generate contrast with ultrasound (US) resolution. Increasingly sophisticated PA setups and signal processing methods are being developed to enhance the capabilities of PA imaging. Phantom experiments are often performed to validate new methods, to characterize PA setups, and to generate training datasets for machine learning. However, the scarcity of high-quality phantoms and the absence of a ground truth in *in vitro* experiments impedes research. Numerical modeling approaches can provide an *in silico* research environment, in which all relevant physical parameters are known and controllable. Therefore, an accurate and validated PA imaging toolchain that can generate realistic PA images may accelerate the general development of PA imaging. However, generating realistic PA image data with simulations remains a great challenge. In this research, we propose a thoroughly validated, quantitative, and flexible PA imaging modeling toolchain and demonstrate its capability to simulate the acquisition of PA signals of a physical PA system in both phantoms and complex tissues.

The fundamental optical and acoustic processes underlying PA imaging are generally well-understood, allowing for physics-based modeling of PA signal generation and acquisition. As this kind of modeling is strictly governed by physical relations, it is inherently transparent and enables full control over the simulated phenomena. Physics-based PA modeling techniques have been used for a wide variety of purposes, e.g., to investigate model-based image reconstruction, to optimize PA setups, and to analyze acoustic effects and artifacts, such as speed of sound aberrations and clutter.^1^^–^^5^ Another emerging research area in which the simulation of data plays a major role is the development of machine learning to improve or interpret measured PA data. Machine learning has the potential to increase the quality of PA images by supressing clutter and noise, to overcome limited-view and limited-bandwidth artefacts, and to provide quantitative estimates of the tissue’s optical and acoustic properties.^6^ However, machine learning is a data-driven approach, and a major bottleneck in its application is the lack of reliable experimental training data and the ground truth. The use of accurate numerical models is therefore essential to generate reliable training datasets in machine learning.^7^

In biomedical applications, the duration of the optical pulses used is typically short enough to meet the thermal and stress confinement criteria needed for adequate optical energy delivery and image resolution.^8^ When these criteria are met, the modeling of PA signals is generally subdivided into an optical part and an acoustic part. First, the locally resolved pressure rise, caused by the absorption of laser light and thermoelastic expansion of the tissue, is determined. To calculate the initial pressure, the optical fluence, which is physically governed by the radiative transfer equation (RTE), needs to be determined. Solving the RTE in light–tissue interaction is done either with statistical Monte-Carlo (MC) methods or using a diffusion approximation of the RTE, which can be solved with continuum methods (e.g., finite-difference and finite element methods).^9^^–^^11^ Next, the propagation of the pressure waves must be simulated by solving the acoustic wave equations. Many different mathematical techniques and software packages to model wave propagation of medical US have been described in the literature, often relying on spectral methods or the finite element method.^12^ For efficient and accurate PA wave propagation modeling and reconstruction in heterogeneous media, the open source toolbox k-Wave is frequently used in the literature.^7^^,^^13^^,^^14^

To be used as a reliable tool to generate realistic PA data, a PA imaging toolchain must be flexible enough to adapt to various setups and media. Typical PA setups can consist of a range of laser sources, e.g., fiber lasers,^15^ crystal lasers,^16^ and diode lasers,^17^^,^^18^ and different types of the acoustic sensors, including single element transducers,^19^ linear array transducers,^18^ and custom-designed transducers.^20^^,^^21^ Furthermore, the tissues on which PA imaging is performed, are often heterogeneous and induce both optical and acoustic scattering, reflections, refraction, and/or attenuation.

The coupling of the optical and acoustic domains, as well as a correct description of both the PA setup and the medium to be imaged are nontrivial tasks. A thorough analysis of the quality of the simulated PA data is needed to ensure realistic results. These analyses, however, are often qualitative or rely on idealized setups and/or simplified media, such as wire phantoms.^4^^,^^7^^,^^14^^,^^22^^–^^25^ A thorough validation of the entire modeling chain with physical PA set-ups in complex media is still missing. This lack of validation makes the accuracy and the realism of the simulations an uncertainty and thus hinders the translation toward *in vivo* applications.

Therefore, in this study, a toolchain is developed and validated by comparing simulated PA images with those acquired by an experimental PA imaging setup. To further improve the quality of the simulated images, this study also investigates the performance of a sampling strategy for the definition of the initial pressure field prescription in k-Wave. The toolchain is designed to be quantitative, by simulating channel data in an absolute unit (Pascal) with a physical model. Furthermore, it was made to be as flexible as possible with respect to the medium geometry and PA system specifications. Both the optical and acoustic parts of the toolchain are validated, and its capability to simulate realistic PA images is demonstrated using multiple *in vitro* phantoms and *ex vivo* human tissue samples of carotid plaques.^26^

## 2 Methods

### 2.1 Optical Fluence Modeling

A grid-based MC software was developed to simulate the optical fluence distribution in complex media. The tool is implemented in C++, callable from MATLAB (R2021a, The Mathworks, Natick, Massachusetts) and compiled with multithreading support using OpenMP (GCC 8.1, Free Software Foundation, Boston, Massachusetts). The implementation is based on the microscopic Beer–Lambert law method, which shows an overall superior convergence rate compared with other methods.^27^^,^^28^ In this method, photon packets with energy $E_{p}$ are tracked that deposit energy in the medium in a continuous way:

$$E_{d}=E_{p}\cdot (1-e^{-\mu_{a}\cdot L_{a}}),$$ (1)

where $E_{d}$ is the energy (J) deposited to a voxel in the medium, $L_{a}$ is the distance (m) traveled by the photon packet in that voxel, and $\mu_{a}$ is the optical absorption coefficient ($m^{-1}$). Energy conservation is maintained by subtracting $E_{d}$ from $E_{p}$ after each deposition. The distance traveled between two scattering events,$\;L_{s}$ (m), is determined as follows:

$$L_{s}=-\frac{\log(\eta )}{\mu_{s}},$$ (2)

where $\mu_{s}$ denotes the scattering coefficient ($m^{-1}$) and η is a random number generated from a uniform probability distribution between 0 and 1. The direction of the photon packet is changed in a scattering event according to the Henyey–Greenstein phase function:

$$p(\theta )=\frac{\left(1-g^{2})\cdot \sin(\theta \right)}{2\cdot (1+g^{2}-2\cdot g\cdot \cos(\theta ){)}^{\frac{3}{2}}},$$ (3)

where g is the scattering anisotropy (–), θ is the angular deflection with respect to the photon packet’s previous direction (rad), and p(θ) is its corresponding probability function.

The optical properties for each grid point in the domain ($\mu_{a}$, $\;\mu_{s}$,   g) can be set according to the local simulated tissue compositions, allowing for a simulation of the photon distribution in complex heterogeneous media.

### 2.2 Acoustic Simulation and Sensor Definition

The propagation of the initial pressure field in the tissue over time is simulated according to the linear wave equation using the k-Wave toolbox. k-Wave is an open source toolbox that uses a pseudospectral, time-domain, finite difference method to solve the linear wave equation for compressional waves.^13^ The largest supported frequency in the simulation is limited by the Nyquist criterion, which states that a sampling of at least two points per wavelength (PPWL) is needed. The largest allowed spacing in the simulation is therefore related to the maximum frequency that the US probe can detect. All waves with a higher frequency will not be sensed by the probe and can thus be safely neglected in the simulation.

To define a sensor in k-Wave, each transducer element must be discretized to pixel positions and associated weights. The position of an element is usually mapped to the grid using nearest-neighbor interpolation.^13^ This approach, however, alters the size and position of the elements and the kerf between them. Furthermore, it causes staircasing artifacts when the elements are not aligned with the grid, negatively affecting the simulated channel data. These artifacts can be reduced by band-limiting the shape of the elements first, using a numerical convolution with the band-limited interpolant (BLI), which gives the correct quadrature weights for discretization.^29^ In this study, an analytical description of the band-limited rectangular element shape was used. The shape was prescribed directly in the spatial Fourier domain using a sinc function, which gave the appropriate quadrature weights after applying the inverse Fourier transform.

### 2.3 Initial Pressure Definition

The Nyquist criterion needs to be considered when sampling $p_{0}$ for use in k-Wave. If the maximum spatial frequency of the initial pressure is higher than the Nyquist frequency, aliasing occurs when sampling this distribution. For initial pressure distributions of structures not aligned with the grid, this effect may appear as staircasing artifacts (pixelated/discontinuous edges) in the reconstructed image.

To avoid aliasing errors, an analytical description of the initial pressure in the spatial frequency domain may be used. The initial pressure field can subsequently be sampled in the frequency domain, which prevents the occurrence of aliasing. This approach, however, is limited to spatial pressure distributions for which analytical descriptions exist.

A more generic and straightforward method would be to use a finer spatial sampling of $p_{0}$ and adjust the acoustic simulation grid accordingly. This method will be referred to as the direct simulation method. Although a direct simulation will converge to the correct solution for decreasing $\Delta x_{\text{sim}}$, it is not viable for most purposes, as it increases computation time significantly, especially for two-dimensional (2D) and three-dimensional (3D) simulations.

Wise et al.^29^ introduced a technique in which the band-limited pressure field is obtained by a numerical integration of point sources, using the BLI. Although this method is theoretically valid for all geometrical shapes, finding the optimal spatial distribution of point sources for arbitrary shapes is a nontrivial task. Furthermore, the BLI has an infinite support, which makes this method computationally demanding. To reduce computation time, truncation of the BLI was suggested at the expense of accuracy.

**Fig. 1:**
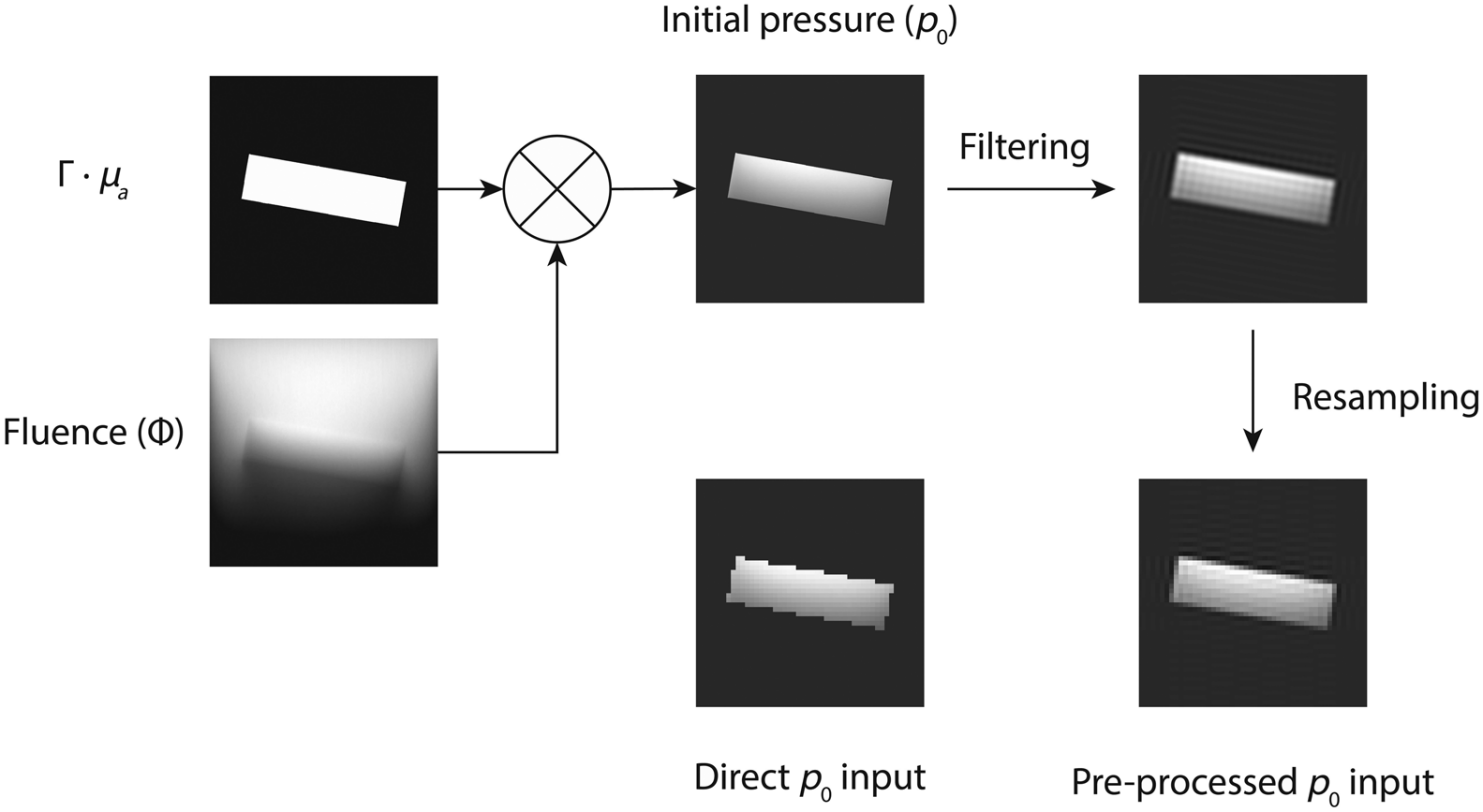
A schematic example of the proposed $p_{0}$ sampling strategy of a rectangular absorber. After determining the optical fluence, $p_{0}$ is calculated using a fine grid spacing. A filtering and resampling step is performed subsequently to sample $p_{0\mathrm{sim}}$ on the coarse grid used in the acoustic simulation.

In this study, we propose a computationally inexpensive and accurate preprocessing step to reduce aliasing artifacts, see Fig. 1. The initial pressure (in Pa) is calculated according to

$$p_{0}=\Gamma \cdot \mu_{a}\cdot \Phi ,$$ (4)

where Γ denotes the Grüneisen parameter [–], $\mu_{a}$ is the optical absorption coefficient ($m^{-1}$) and Φ is the optical fluence ($J/m^{2}$) as determined by the optical MC simulation. Unlike the relatively large spatial spacing used for the acoustic simulation, $\Delta x_{\text{sim}}$, a smaller spacing spatial spacing $\Delta x_{\text{pre}}$ ($\Delta x_{\text{pre}}<\Delta x_{\text{sim}}$) is used to sample $p_{0}$. This smaller $\Delta x_{\text{pre}}$ allows for a better sampling of $p_{0}$, as less content in the spatial frequency domain is aliased.

To create speckle in the PA images, a Gaussian distribution was used to model deviations in the absorption coefficient and Grüneisen parameter. In this model, random fluctuations were added to the initial pressure:

$$p_{0_{\mathrm{speckle}}}=p_{0}+N(\sigma_{\mathrm{speckle}})\cdot \Phi ,$$ (5)

$$\sigma_{\mathrm{speckle}}=\sqrt{I_{\mathrm{speckle}}}\cdot (\Delta x_{\mathrm{pre}}{)}^{-\frac{D}{2}},$$ (6)

where N(σ) denotes a zero mean normal distribution with standard deviation σ, $I_{\mathrm{speckle}}$ is a spatially distributed quantity proportional to the local acoustic speckle intensity, and D is the number of simulated dimensions (D=1,2,3, corresponding to 1D, 2D, and 3D simulations) in the acoustic simulation. The correction factor ${\left(\Delta x_{\mathrm{pre}}\right)}^{-(D/2)}$ ensures the simulated speckle intensity is independent of the used sampling spacing, see Appendix A.

**Fig. 2:**
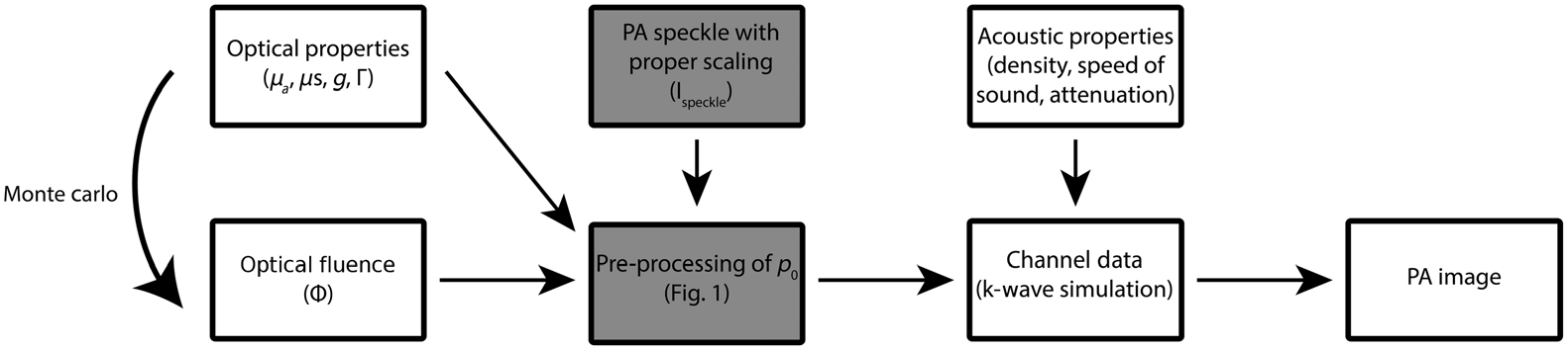
A schematic overview of all processing steps. The gray boxes indicate enhanced processing methods proposed in this study.

Next, $p_{0_{\mathrm{speckle}}}$ is filtered with a multidimensional ideal low-pass filter H to remove frequencies higher than the maximal frequency supported in the simulation:

$$\mathrm{rect}(\xi )=\{\begin{array}{ll} 1 & \text{for }|\xi |\leq \frac{1}{2}\\ 0 & \text{for }|\xi |>\frac{1}{2} \end{array},$$ (7)

$$H(\overset{\to }{k})=\overset{D}{\underset{i=1}{\prod }}\mathrm{rect}(k_{i}\cdot \Delta x_{\mathrm{sim}}),$$ (8)

$$p_{0_{\mathrm{speckle}}}^{*}=F^{-1}\{F(p_{0_{\mathrm{speckle}}})\cdot H(\overset{\to }{k})\},$$ (9)

where F denotes the multidimensional spatial Fourier transform and $\vec{k}$ spatial frequency. Finally, the filtered distribution $p_{0_{\mathrm{speckle}}}^{*}$ is downsampled to obtain $p_{0\mathrm{sim}}$, which was prescribed as input in k-Wave. A schematic overview of all processing steps in the toolchain, is shown in Fig. 2.

### 2.4 Staircasing Error Reduction Analysis

To estimate the performance of the method proposed, both the required computation time and the error in the delay-and-sum reconstructed PA images of disc-shaped $p_{0}$ distributions, with a radius of 1 and 5 mm, were determined. The simulations were performed on a PC with an i7-10700 processor (Intel, Santa Clara, California) and an RTX 3070 GPU (Nvidia, Santa Clara, California), using the optimized CUDA k-Wave code. The $p_{0\mathrm{sim}}$ distributions were obtained using three sampling strategies: (1) the preprocessing method proposed with a fixed $\Delta x_{\text{sim}}$ and a varying $\Delta x_{\text{pre}}$, (2) by means of the Wise et al. method^29^ with a fixed $\Delta x_{\text{sim}}$ and a varying number of integration points $N_{\mathrm{int}}$, and (3) using a direct simulation with varying $\Delta x_{\text{sim}}$. The kWaveArray class (alpha version 0.3) was used as implementation of the Wise et al. method, and the default truncation of the BLI at 5% was applied. In this implementation, the disc is sampled in a radial pattern, with the number of integration points increasing linearly as a function of the radial position to enhance uniformity. For comparison, $N_{\mathrm{int}}$ was expressed as a function of the effective sampling distance $\Delta x_{\mathrm{eff}}$ using the following equation:

$$N_{\mathrm{int}}=\frac{A_{\mathrm{obj}}}{(\Delta x_{\mathrm{eff}}{)}^{2}},$$ (10)

where $A_{\mathrm{obj}}$ denotes the area ($m^{2}$) of the simulated object.

The simulation results were compared with an analytical solution of a low-pass filtered disc phantom that exhibits no staircasing. This reference PA phantom was obtained by means of a simulation in which $p_{0\mathrm{sim}}$ was sampled in the spatial frequency domain:

$$p_{0_{\mathrm{sim}}}=F^{-1}(\frac{J_{1}(2\pi \sqrt{\xi_{x}^{2}+\xi_{y}^{2}})}{\sqrt{\xi_{x}^{2}+\xi_{y}^{2}}}),$$ (11)

where $\xi_{x}$ and $\xi_{y}$ denote the spatial frequency (scaled with respect to the radius) in x and y directions, respectively, and $J_{1}$ is the order-1 Bessel function of the first kind.

The error ε, with respect to the reference simulation, was calculated using

$$\varepsilon =10\cdot \log_{10}\frac{\sum (p-p_{\mathrm{ref}}{)}^{2}}{\sum p_{\mathrm{ref}}^{2}},$$ (12)

where p and $p_{\mathrm{ref}}$ denote the pressures of the reconstructed radio frequency signals, respectively.

### 2.5 In Vitro and In Silico Experimental Setup

**Fig. 3:**
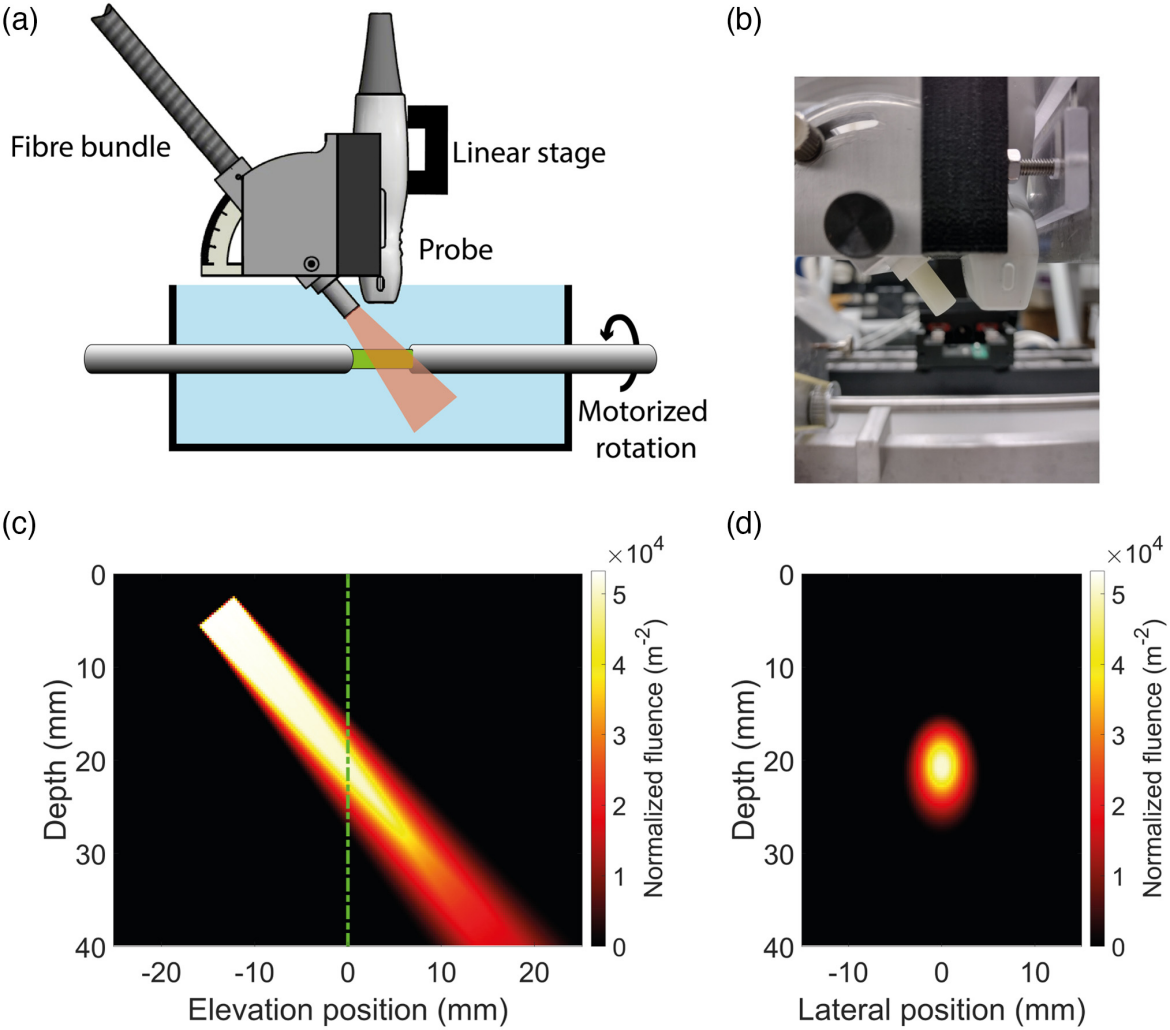
(a) A schematic drawing of the setup used. The fiber bundle and probe are attached to a linear stage using the probe holder. The mounted sample can be rotated by a motor. (b) A photograph of the probe holder. (c) A simulation of the normalized fluence ($m^{-2}$) in water. The probe’s imaging plane is indicated by the dashed line. (d) The optical fluence in the probe’s imaging plane, corresponding to the green dashed line in (c).

The developed toolchain was validated with a real experimental setup. The setup consisted of a tunable pulsed laser (Opotek, Radiant HE 355LD, Carlsbad, California) with a pulse duration of 6 ns and a fiber bundle with a custom designed circular output aperture of 4.9 mm in diameter (CeramOptec, Bonn, Germany). A Vantage 256 system (Verasonics, Kirkland, Washington) connected to a Verasonics L11-5v linear array transducer with 128 elements, a pitch of 300  μm, and an elevational focus depth of 18 mm, was used as US detector. The probe’s center frequency was 7.6 MHz, and it had a bandwidth of 75%. The frequency response of the probe was implemented in the toolchain using a linear frequency filter, defined as a Hann window in the temporal Fourier domain. The center frequency and bandwidth of the Hann filter (at −6  dB) in the simulations were chosen to match the probe used. The pixel spacing used in k-Wave, $\Delta x_{\text{sim}}$, was set to 30  μm, unless stated otherwise. The fiber bundle and probe were aligned and mounted to a linear stage using a custom-designed probe holder, see Figs. 3(a) and 3(b).^30^ In the optical simulations, the fiber bundle tip was approximated as a disc source with uniform intensity. The angular distribution of the emitted photons was uniform within a cone with a maximum angle with respect to the bundle’s surface of 5 deg, see Figs. 3(c) and 3(d). All optical simulations were performed in 3D with a grid spacing of 250  μm. The medium properties were constant in the elevational direction. The acoustic simulations, however, were performed in 2D in the imaging plane of the linear probe. Acoustic attenuation was not considered, as the acoustic attenuation is relatively insignificant compared with the effective optical attenuation for all experiments.

### 2.6 Verification of Simulated PSF in Turbid Media

The ability of the toolchain to accurately simulate the point spread function (PSF) in turbid media was verified experimentally. A black thread (280  μm diameter) served as an absorber and was positioned in a tank filled with deionized water. The angle between the normal direction of the probe and the laser source (800 nm) was 40 deg. The position of the probe and the laser source could be adjusted simultaneously using a linear stage connected to the probe holder, to vary the distance to the black thread in steps of 0.5 mm, see Fig. 3. The reconstructed pressure at the thread’s position in the PA images was determined for each position and were compared between the *in vitro* and simulated results.

Intralipid (Intralipid 20%, Fresenius Kabi Nederland, Huis Ter Heide, The Netherlands) was added to the water to increase the scattering coefficient. The 20% intralipid was diluted to solutions with a range of five concentrations (0.01%, 0.02%, 0.05%, 0.1%, and 2%). This range of concentrations corresponds to reduced scattering coefficients between 0.1 and $2\;{\mathrm{cm}}^{-1}$.^31^ As the absorption of intralipid at 800 nm wavelength is negligible, only absorption due to the water ($\mu_{a}=2.3\;m^{-1}$) was taken into account in the simulations.^31^^,^^32^ The scattering anisotropy factor was set to 0.55, in agreement with estimations of the anisotropy factor of intralipid using Mie theory^31^.

### 2.7 Three-Channel Phantom

**Fig. 4:**
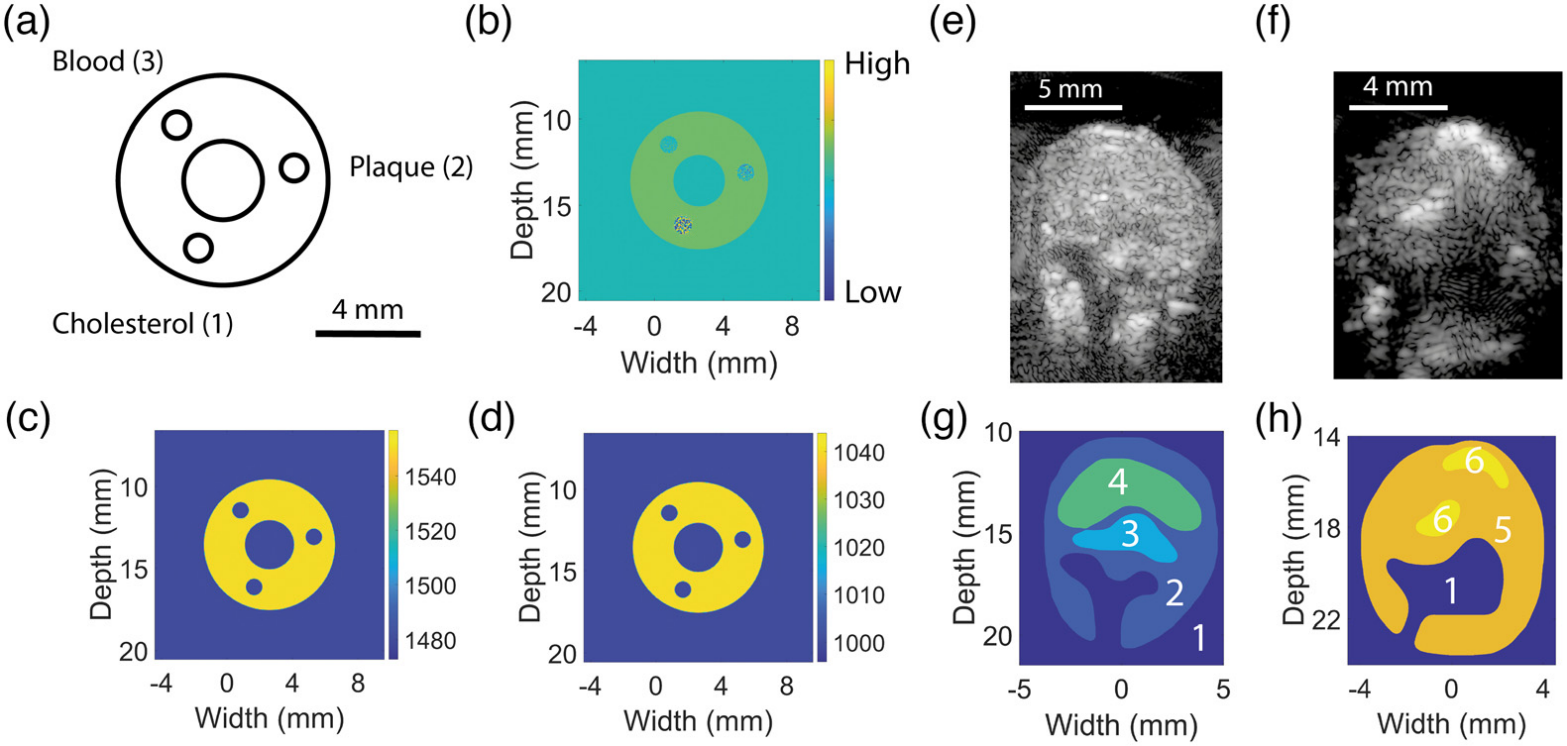
The properties of the three-channel phantom used in the toolchain validation. (a) The three channels are filled with cholesterol, a human plaque sample, and blood, respectively. (b) The initial pressure field, (c) the speed of sound map, and (d) the density map as prescribed in the acoustic simulation. (e) and (f) Two *in vitro* US images of human plaque samples. The corresponding segmentations used in the simulations are shown in (g) and (h).

To assess the performance of the acoustic simulation in reproducing the visual appearance of realistic tissues, a controlled *in vitro* study was conducted using a polyvinyl alcohol (PVA) phantom with three channels representing plaque in a carotid artery, see Figs. 4(a)–4(d).^33^ The three channels were filled with: (1) cholesteryl linoleate (Sigma-Aldrich, St. Louis, Missouri), (2) pieces of a human plaque, and (3) porcine blood collected from the slaughterhouse with 19% w/v sodium citrate tribasic dihydrate (Sigma-Aldrich, St. Louis, Missouri) as anticoagulant. The human plaque samples in this study were obtained during a carotid endarterectomy as part of an *in vivo* PA imaging study previously reported by our group.^26^ The phantom was fixed in a rotatable set-up that allowed for tomographic compounding to increase PAI quality and field of view.^33^ An optical wavelength of 710 nm was chosen to illuminate the phantom, as this resulted in relatively high-quality images with uniform absorption within each channel. The phantom was rotated over 180 degrees in steps of 18 degrees, to apply an even illumination. A uniform fluence distribution was used in the acoustic simulations. Gaussian noise was added to the simulated channel data to match the noise level of the *in vitro* measurements and the Grüneisen parameter was spatially homogeneous and fixed for all simulations. An identical band-pass filter (3 to 13 MHz) was applied to both the simulated and the *in vitro* channel data to remove noise and artifacts beyond the bandwidth of the transducer. Afterward, the channel data were delay-and-sum beamformed to create an image for each rotation angle. The resulting images were spatially compounded, either coherently (averaging of radio frequency data) or incoherently (averaging after envelope detection).^34^ To mimic registration errors in the *in vitro* set-up, the simulated PA images were translated randomly in both axial and lateral direction, using an empirically determined Gaussian distribution with a standard deviation of 200  μm before spatial compounding.

### 2.8 Ex Vivo Plaque Imaging

The developed toolchain was used to simulate *ex vivo* PA images of two human plaque samples to evaluate the performance in a more complex and heterogeneous tissue. The plaques were scanned using an optical wavelength of 800 nm. Manual segmentations of the plaque were made using US B-mode data and histology. Different tissue types were assigned to each region in the plaque, see Figs. 4(e)–4(h). The optical and acoustic properties in the simulation were assigned per region, see Table 1. Note that the tissue types and corresponding material properties for each region were tuned to match the *ex vivo* PA images and may not reflect the ground truth exactly. The. $I_{\mathrm{speckle}}$ and the noise level were tuned empirically to be in agreement with the observed speckle intensity and noise in the *ex vivo* images and the Grüneisen parameter was spatially homogeneous and fixed for both simulations. An identical band-pass filter (3 to 13 MHz) was applied to both the simulated and the *in vitro* channel data.

**Table 1:** The optical (at 800 nm wavelength) and acoustic parameters used in each segmented region of the two plaques. The reference value for $I_{\mathrm{speckle}}$ at 0 dB is $8.9\cdot 10^{-6}$.

Tissue type	Speed of sound (m/s)	Density ($\mathrm{kg}/m^{3}$)	$I_{\mathrm{speckle}\;}$ (dB)	Optical absorption (1/cm)	Optical reduced scattering (1/cm)	Anisotropy factor g (∼)
Lumen/water (1)	1480	1000	−∞	0.023^32^	0^32^	—
Fibrous tissue (2)	1540	1050	−10	0.8^35^	12.6^36^	0.93^36^
Necrotic core (3)	1540	1000	−10	0.8^35^	13.4^36^	0.93^36^
Hemorrhage (4)	1540	1000	−5	1.6^35^	12.6^36^	0.93^36^
Fibrous tissue (5)	1540	1050	0	0.8^35^	12.6^36^	0.93^36^
Calcification (6)	1580	1100	0	1.1^35^	16.4^36^	0.93^36^

## 3 Results

### 3.1 Initial Pressure Preprocessing

**Fig. 5:**
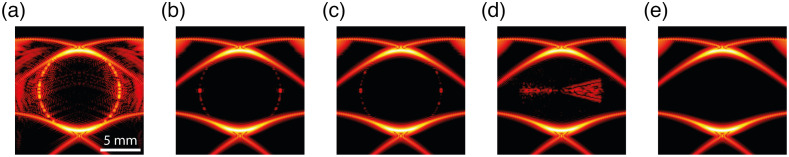
The reconstructed PA images of the 5-mm radius disc are shown (60 dB dynamic range) for different sampling strategies of $p_{0}$: (a) $p_{0}$ was sampled directly on the k-Wave grid with 30  μm spacing (6.6 PPWL); (b) $p_{0}$ was directly sampled on a finer k-Wave simulation grid with 40 PPWL; (c) $p_{0}$ was sampled using the preprocessing method proposed with 40 PPWL; (d) $p_{0}$ was sampled using the Wise et al. method with 40 PPWL. The reference PA image is shown in (e), in which $p_{0}$ was sampled in the spatial Fourier domain.

A simulation of the 5 mm radius disc with the default spatial sampling width $\Delta x_{\mathrm{sim}}$ of 30  μm (6.6 PPWL at the probe’s center frequency) leads to significant aliasing artefacts in the reconstructed image, see Fig. 5(a). Although the top and bottom signals are reconstructed correctly, staircasing artifacts arise in the regions between 1 and 5 o’clock, and between 7 and 11 o’clock. Furthermore, the side lobes and grating lobes of these staircased signals cause an erroneously elevated background signal in a large part of the image. By increasing the PPWL to 40, which results in a $p_{0}$ spatial sampling width of 4.9  μm, the errors could be reduced significantly, at the cost of a strong increase in memory occupation and computation time. The computation time increased from ∼14  s to 36 min between the simulations performed with 6.6 PPWL and 40 PPWL using the direct method. The method by Wise et al.^29^ increased the computation time from 22 s to 4.7 min and showed an erroneously elevated signal within the disc, but no staircasing artifacts at the disc’s boundary. The PA image simulated using the preprocessing method is almost identical to the one simulated with the direct method. Hence, the preprocessing step is effectively reducing aliasing errors. The additional computation time needed for the preprocessing step was 3 s, yielding a total simulation time of 17 s.

**Fig. 6:**
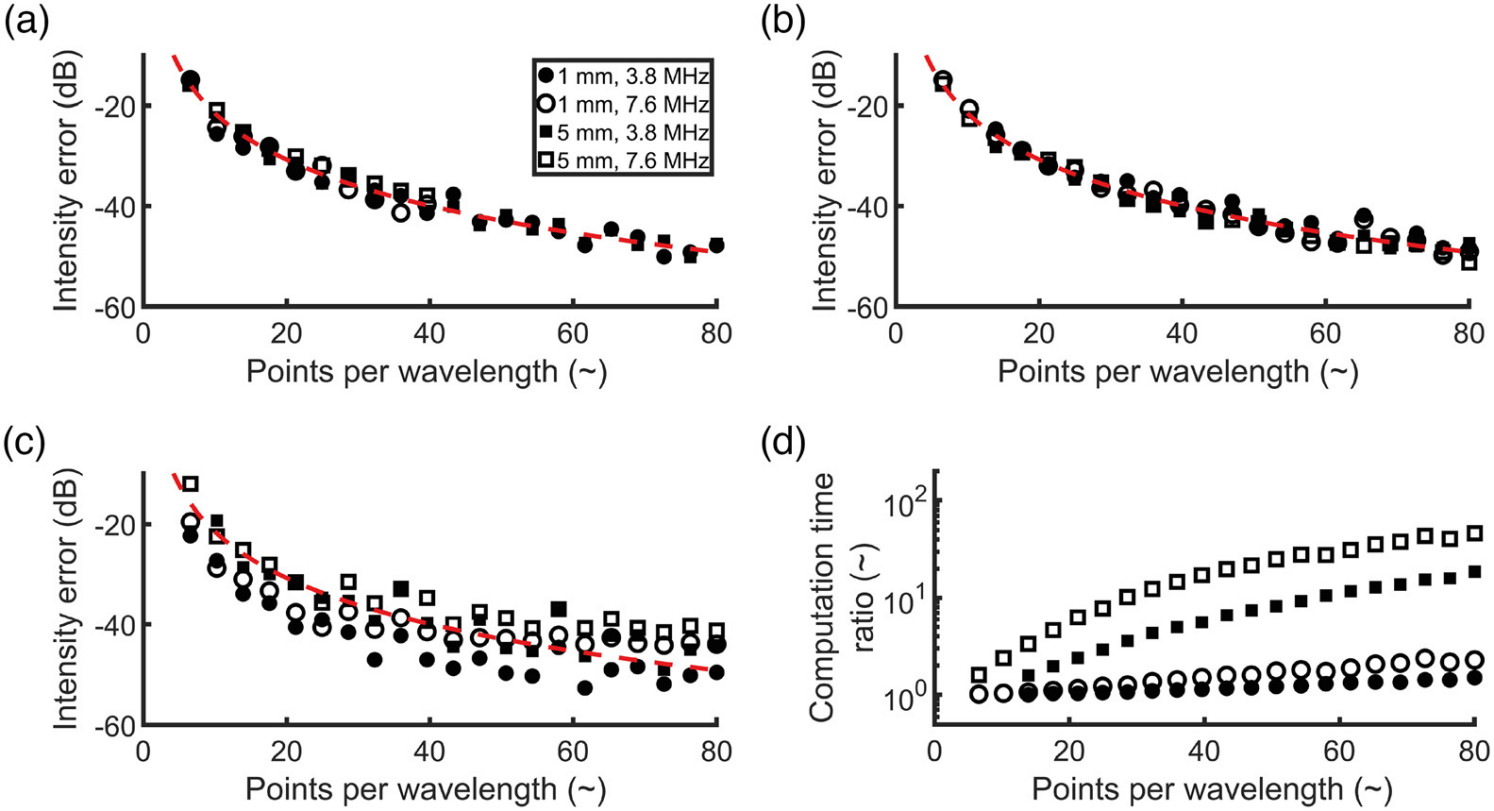
(a) Intensity error ε is shown as a function of the pixel spacing of the acoustic simulation using the direct simulation method. (b) ε is shown as function of $\Delta x_{\text{pre}}$ in the method proposed,^29^ with a constant acoustic simulation pixel spacing of 30  μm. (c) ε is shown as a function of $\Delta x_{\text{eff}}$ in the method by Wise et al., with a constant acoustic simulation pixel spacing of 30  μm. The solid red lines show the fit as described by Eq. (13). (d) The computation time ratio needed for the combined $p_{0}$ sampling and k-Wave simulation between the method by Wise et al.^29^ and the method proposed.

The effectiveness of both the direct method and the preprocessing method was quantified by calculating the error of the PA images, ε, with respect to the results from the reference simulation, see Fig. 6. The reduction of the error is similar between the direct simulation and the preprocessing method, for an equal number of PPWL. For these methods, ε is independent of the size of the disc-shaped $p_{0}$ distribution and the center frequency of the probe and shows algebraic convergence with increasing PPWL. For the method proposed by Wise et al.,^29^ the results show a similar trend, although the intensity error is less similar between different disc sizes and center frequencies, see Fig. 6(c). The simulations using the preprocessing method proposed were generally faster than those using the Wise et al. method, up to 47 times for the large disc at 7.6 MHz center frequency with 80 PPWL, see Fig. 6(d). An equation was fitted empirically to determine the mean error of the reconstructed image as function of the number of PPWL for the preprocessing method proposed:

ε≈−13.3·ln(PPWL)+9.09. (13)

### 3.2 Fluence Simulation in Turbid Medium

**Fig. 7:**
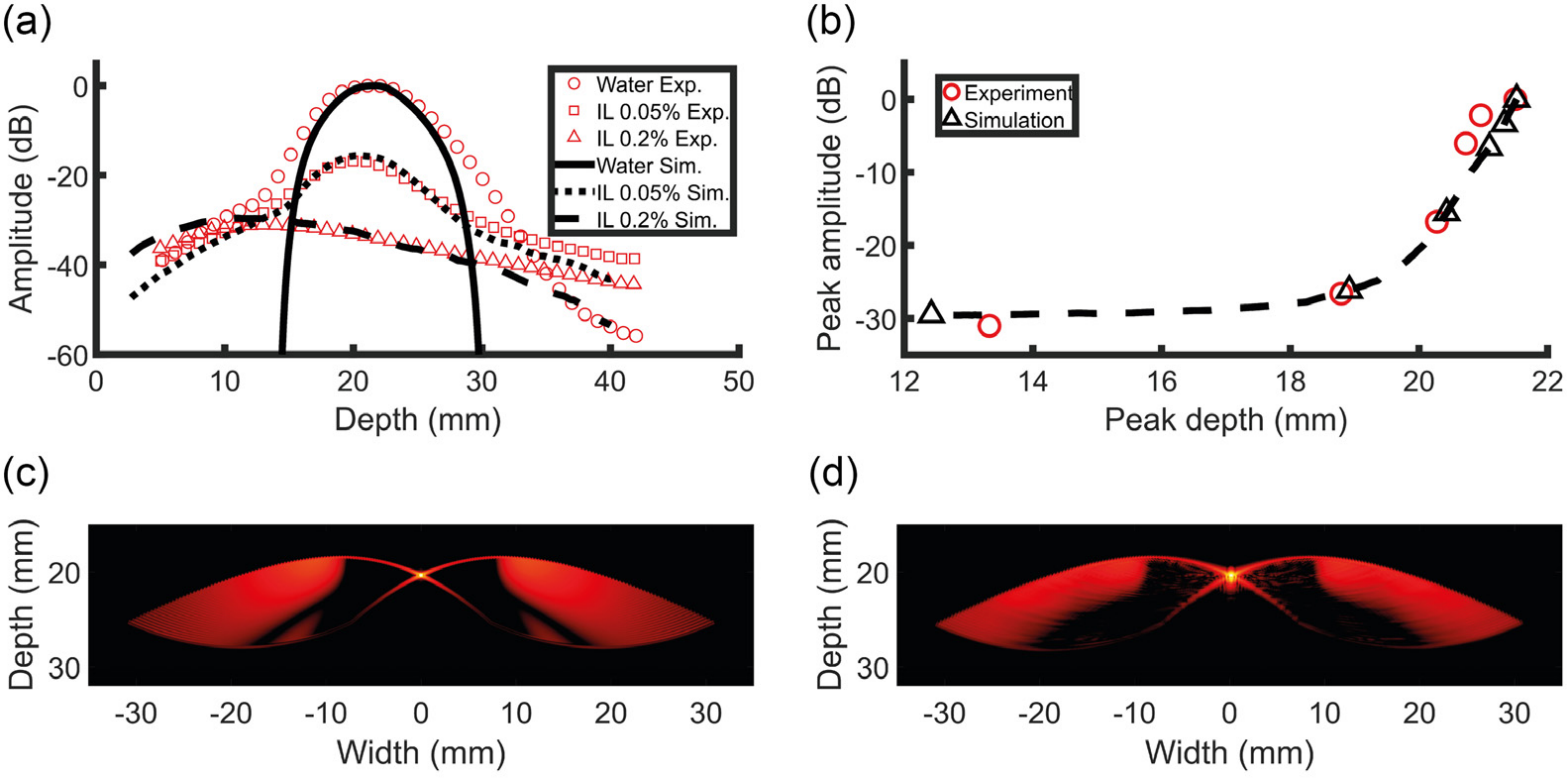
(a) The maximum reconstructed pressure of the black thread phantom is shown for water and two different concentrations of intralipid. (b) The maximum pressures and corresponding depths of the PSF are shown for water and all concentrations of intralipid. The dashed line shows the results for additionally simulated concentrations of intralipid. (c) The simulated PSF in water. (d) The *in vitro* PSF in water. A dynamic range of 60 dB was used for visualization.

The PSF of the black thread was measured *in vitro* and compared with the simulated result, see Figs. 7(c) and 7(d). Overall, the appearances of the *in vitro* and simulated PSF are similar. The axial resolution of the *in vitro* PSF, 310  μm (−6  dB peak width) was slightly worse than the simulated resolution of 270  μm. The *in vitro* and simulated positions of the grating lobes are almost identical, and the lobes’ amplitudes are comparable in general. The amplitude of the reconstructed pressure was measured for a range of intralipid concentrations *in vitro*, see Figs. 7(a) and 7(b). By increasing the intralipid concentration, the reduced scattering coefficient is increased as well, leading to a smaller peak amplitude and a shift of the peak location toward smaller depths. The change in the peak’s amplitude and depth as function of intralipid concentration in the simulated data corresponded well to the experimental results over a relatively large range of pressure amplitudes (30 dB).

### 3.3 Three-Channel Phantom

**Fig. 8:**
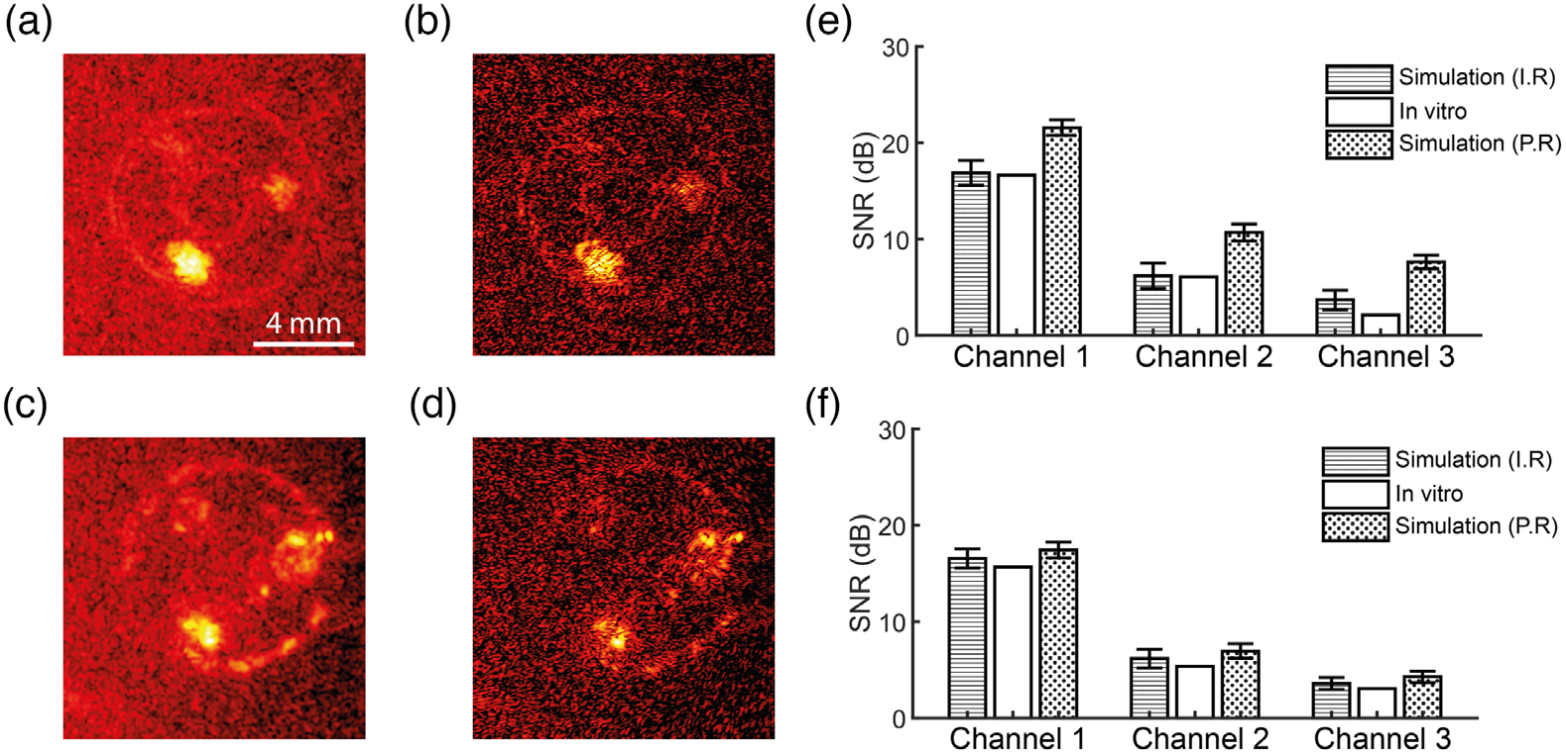
(a), (b) The simulated PA images for incoherent and coherent spatial compounding. (c), (d) The *in vitro* PA images for incoherent and coherent spatial compounding. All images are shown with a dynamic range of 30 dB. The SNR (n=10 for simulated data, error bars denote standard deviation) for each channel is shown in (e) for the coherently compounded images, and in (f) for the incoherently compounded images. The SNR of the simulated data was calculated both with an imperfect registration (I.R.) and with a perfect registration (P.R.).

The *in vitro* and simulated PA images of the three-channel phantom were obtained by spatial compounding of the acquisitions over all rotation angels, see Figs. 8(a)–8(d). The *in vitro* and simulated PA images appear to be similar in terms of overall contrast and resolution of the PVA phantom and its three channels for both compounding techniques. The signal-to-noise ratio (SNR) was determined for all channels and compounding techniques, see Figs. 8(e) and 8(f). Overall, the SNR of the coherent compounded simulated images in which no registration errors were added was more than 5 dB larger than the *in vitro* data. The increased SNR was observed in the incoherent simulated images too, although less pronounced. By including registration errors in the simulated data, the mean SNR of all channels was decreased by 4.4±0.39  dB for coherent compounding, and by 0.77±0.10  dB, compared with simulated data with perfect registration. After adding registration errors, the SNR of simulated data agreed well with the *in vitro* data for all channels and for both compounding techniques. The mean SNR decrease for coherent compounding was 3.6 dB larger than the decrease for incoherent compounding. This observation can be explained by the fact that coherent averaging is more susceptible to registration errors than incoherent compounding, as the latter does not take phase information into account.

### 3.4 *Ex Vivo* Plaque Imaging

**Fig. 9:**
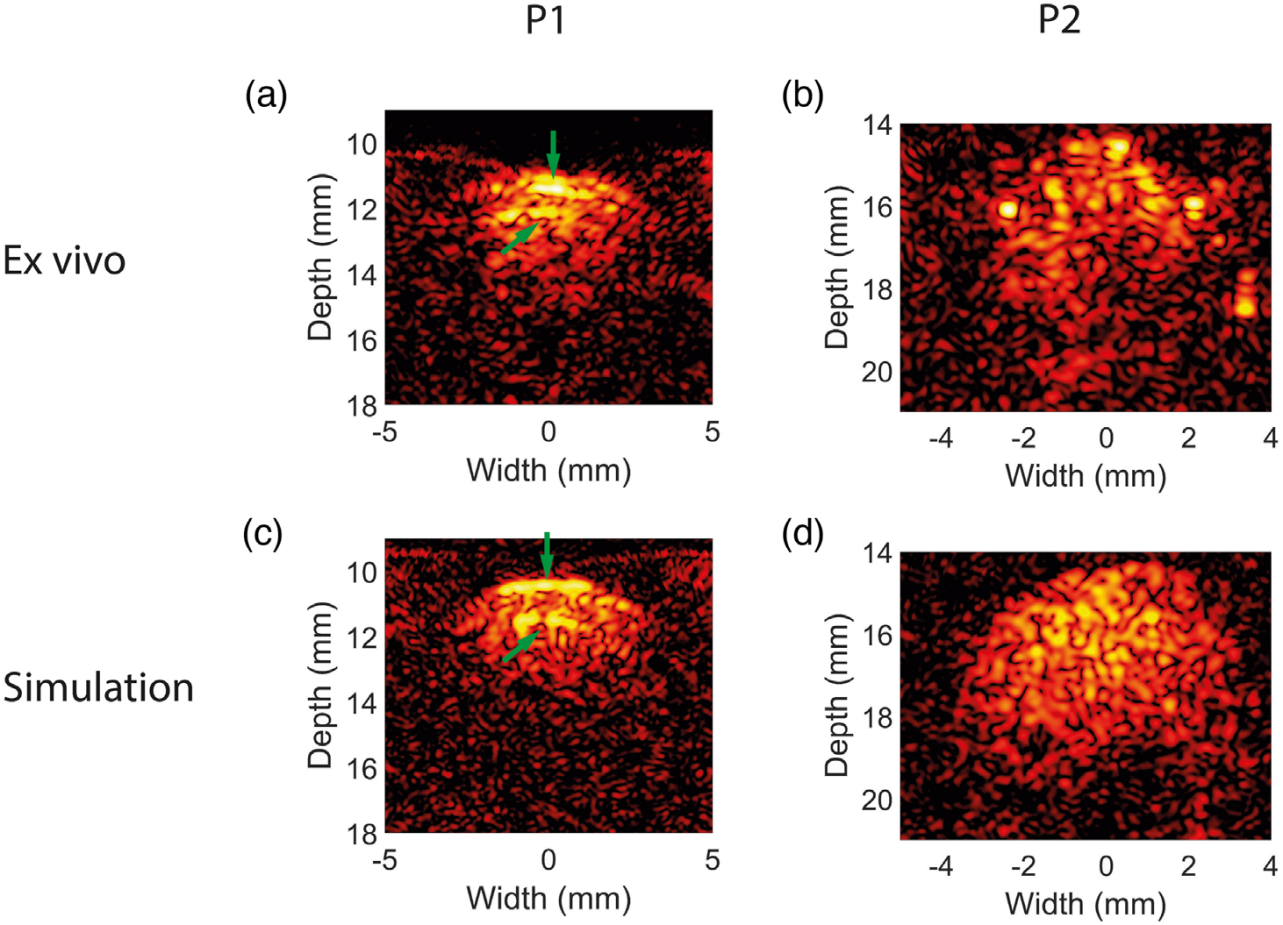
(a), (b) Two *ex vivo* PA images (at 800 nm) of different human plaques (P1, P2). (c), (d) The corresponding simulations. Two PA signals originating from the interface between two absorbing layers can be observed in (a) and (c) and are indicated by the arrows. The dynamic range of all images is 35 dB.

Two human carotid plaques were scanned *ex vivo* at a wavelength of 800 nm, see Figs. 9(a) and 9(b). The top parts of the plaques show relatively strong speckle signals. The intensity of these signals drops rapidly over only several millimetres deeper into the plaque, implying a strong fluence decrease with increasing depth. Furthermore, two PA interface signals originating from sharp transitions in the local absorption coefficient can be observed in the first plaque, see Fig. 9(a). These signals, indicated by the green arrows, appear only as lines due to the band-limitation of the probe.^26^ Overall, the simulated PA images agree well with the *ex vivo* images in terms of overall appearance, resolution, and contrast, see Figs. 9(c) and 9(d). Furthermore, both the rapid fluence drop as function of depth, and the two PA interface signals could be simulated realistically. However, the top region of the plaque in Fig. 9(b) shows several high-intensity absorbers that were not present in the used segmentation. The Gaussian model for speckle could not simulate these high-intensity absorbers.

## 4 Discussion

Many numerical methods to simulate the acoustic wave propagation and the optical absorption in tissue have been described and verified before, using both analytical solutions and *in vitro* experiments.^11^^,^^12^^,^^37^^,^^38^ However, a thorough validation of the entire modeling chain with physical PA setups in complex media is still missing. This lack of validation makes the accuracy and the realism of the simulations an uncertainty and thus hinders the translation toward *in vivo* applications. In this study, a modeling toolchain has been developed and thoroughly validated to simulate the acquisition of PA signals from complex media with a physical PA imaging system. The toolchain is based on two separate modeling steps: simulating the initial pressure field first and subsequently simulating the acoustic propagation of the generated PA waves. Furthermore, a new sampling strategy for the initial pressure was proposed to reduce discretization artifacts in the reconstructed images. The toolchain developed allows for quantitative simulations of PA signals, with a proper scaling factor for speckle intensities. The toolchain was designed to be as generic as possible, such that many different types of PA systems and media can be simulated.

The optical fluence was calculated by means of an MC method, which enables the simulation of the light distribution in complex media according to the RTE. A grid-based approach was taken to simulate the optical fluence, as this allowed for efficient raytracing of the photon packets and a convenient interpolation to the grid-based acoustic wave field solver of k-Wave. The developed MC software employs the microscopic Beer–Lambert law method for faster convergence and is parallelized for improved performance compared with traditional MC codes.^28^^,^^39^ Although more sophisticated methods have been described in the literature that make use of graphical processing units to increase computational performance,^40^ the developed software was mainly aimed to offer a practical and user-friendly modeling platform. A drawback of the used grid-based method, however, is a less accurate simulation of refraction and reflection effects, compared to mesh-based methods.^41^^–^^43^ The effect of refraction and reflection was deemed relatively small compared with scattering and absorption effects in most biological tissues.

The acoustic wave modeling software k-Wave was used in the toolchain to simulate the propagation of PA waves. k-Wave solves the wave equations governing the behavior of PA signal propagation through media and is therefore able to model all relevant acoustic phenomena, i.e., second and higher order scattering, frequency-dependent attenuation, reflection, and refraction. Due to the grid-based nature of k-Wave, staircasing occurs when discretizing the initial pressure field. Staircasing is a fundamental issue in grid-based discretization and has been studied in other physical domains as well, for example, in electromagnetics.^44^ The effect of staircasing in band-limited pressure fields in PA was investigated in this study. The numerical method to prescribe $p_{0}$ in k-Wave was optimized to suppress staircasing errors in the simulated PA data. The preprocessing proposed step can achieve a similar performance as the direct simulation method (finer simulation grid) and the method introduced by Wise et al. (integration of point absorbers) in reducing the reconstructed intensity error, by decreasing the spatial spacing only when calculating *p*_0_ and leaving k-Wave’s grid spacing intact. The staircasing artifacts at the disc’s boundary were absent in the Wise et al. method. Therefore, the Wise et al. method preserves the geometrical shape of the absorbers better, even at smaller sampling spacings. Still, it suffered from other artifacts in the region within the disc. These artifacts are probably caused by the BLI thresholding at 5% and by a slight nonuniform distribution of the integration points within the disc. Both the Wise et al. method and the direct simulation method led to a significant increase in computation time, as they were ˜17 and 125 times slower than the preprocessing method, respectively, when simulating a 5-mm radius disc at a $p_{0}$ sampling of 40 PPWL. Note that the acoustic simulations were performed in 2D and that the required simulation time for the methods can differ in 3D. The preprocessing step therefore effectively improves the dynamic range of the PA images that can be simulated accurately. Furthermore, the preprocessing step is performed independent of the actual optical and acoustic simulations, allowing it to be used effectively with other methods and software tools than used in this study. To ensure a convenient workflow for the user, the optimal grid spacing in the preprocessing method can be straightforwardly determined using an empirical fit [Eq. (13)], after specifying the required dynamic range in the simulated PA images.

To validate the toolchain, simulated images of two phantoms and an *ex vivo* carotid plaque were compared with reconstructed images from a real PA set-up. The validation was performed on PA images, as this is a convenient and natural way to process and analyze the data. However, as the toolchain provides the raw channel data, the processing of the data is not limited to imaging only. Although all simulation results agree well with the real PA measurements in general, some small deviations are still present. The axial resolution of the simulated PSF was close to the *in vitro* measured resolution with only an absolute error of 40  μm (0.2 wavelengths) as measured by the FWHM of the wire signal. The underestimation of FWHM of the wire in the simulation may be caused by the relatively large diameter of the wire (280  μm) which is larger than the wavelength at the probe’s center frequency (200  μm). Still, the simulated grating lobes agreed well with the experimental data in terms of reconstructed intensity and position. In this study, the system’s frequency response filter was defined by means of the probe’s center frequency and bandwidth using a Hann window in the temporal frequency domain. Although this approach is relatively straightforward and accurate for the *in vitro* system used, the PSF could be further improved by measuring the impulse response using a point absorber much smaller than the acoustic wavelength or directly using a calibrated hydrophone.

The simulated pressure amplitude and depth of maximum PA signal intensity in the reconstructed images matched well with the *in vitro* data for all concentrations of intralipid. The simulated pressure profiles as function of depth were accurate, although the pressure amplitude was generally underestimated in regions next to the peak. As this error is also present in the case of 0% intralipid, the error is probably caused by an inaccurate simulation of the light source. Furthermore, optical reflections in the *in vitro* set-up may also contribute to the discrepancy between the simulated and *in vitro* profiles. Moreover, it should be noted that, in the simulations, the Henyey–Greenstein phase function was used to calculate the optical scattering angle distribution, as is often done in tissue optics, whereas the scattering in intralipid is dominated by Mie scattering. These different phase functions may change the appearance of the pressure profiles slightly, even for equal reduced scattering coefficients and scattering anisotropy factors. Still the results suggest that simulations can replicate the actual optical wave propagation with high accuracy.

The PA speckle present in the *ex vivo* images was simulated using a Gaussian model to add random fluctuations to the initial pressure field. The appearance of PA speckle patterns in the simulated images, however, is highly dependent on the segmentation and prescription of the initial pressure. Although the Gaussian model gives reasonable results, other distributions or techniques to define the initial pressure may be investigated to increase the realism of the simulated PA signals in tissue models.

## 5 Conclusion

In this study, a flexible toolchain to simulate PA signals has been developed and validated. MC software was developed to determine the optical fluence in the illuminated medium and the open-source k-Wave toolbox was used to simulate the acoustic propagation of the pressure field over time. A computationally efficient preprocessing step was developed to decrease aliasing errors in the prescribed initial pressure field, which cause incorrect signals in the reconstructed PA images. It was shown that this preprocessing step could achieve a similar reduction in aliasing errors compared to the traditional approach of decreasing the spatial step in the k-Wave simulation. The toolchain was validated by *in vitro* measurements of a black wire, a three-channel phantom and *ex vivo* images of two human carotid plaques. It was shown that the PSF could be simulated accurately in turbid media. The overall appearance and SNR of the simulated PA images of the three-channel phantom and the two plaques showed good agreement to real PA measurements, indicating the great potential of the PA simulation in a more complex and heterogeneous media using the proposed toolchain.

## 6 Appendix

The energy of a 1D signal can be expressed using the following equation:

$$E=\sum_{n=0}^{N-1}s(n{)}^{2}\;\Delta x,$$ (14)

where s(n) is the signal at position n, and Δx is the spatial pixel spacing.

For a zero mean noise signal, the expected energy can also be written as function of the variance of s:

E=N·Var(s)·Δx. (15)

To simulate a constant speckle intensity for all possible Δx, the energy per unit length must be constant. Furthermore, because the total power of white noise scales proportionally with the bandwidth, the energy per unit of frequency should also be constant. $\;I_{\mathrm{speckle}}$ can be related to E by applying a proper scaling:

$$I_{\mathrm{speckle}}=\frac{E}{L\cdot f_{s}}=\frac{E}{(\Delta x\cdot N)\cdot \frac{1}{\Delta x}}=\frac{E}{N}=\mathrm{Var}(s)\cdot \Delta x,$$ (16)

where L is the total length of the signal and $f_{s}$ is the spatial sampling frequency. Finally, $I_{\mathrm{speckle}}$ can be related to the standard deviation of s, $\sigma_{s}$:

$$\sigma_{s}=\sqrt{\mathrm{Var}(s)}=\sqrt{\frac{I_{\mathrm{speckle}}}{\Delta x}}.$$ (17)

The derivation can be generalized for multiple dimensions to obtain Eq. (6).

## Code, Data, and Materials Availability

The code and data underlying the results presented in this paper are not publicly available at this time but may be obtained from the authors upon reasonable request.
